# Chronic inflammation markers and cytokine-specific autoantibodies in Danish blood donors with restless legs syndrome

**DOI:** 10.1038/s41598-022-05658-1

**Published:** 2022-01-31

**Authors:** Joseph Dowsett, Maria Didriksen, Jakob Hjorth von Stemann, Margit Hørup Larsen, Lise Wegner Thørner, Erik Sørensen, Christian Erikstrup, Ole Birger Pedersen, Morten Bagge Hansen, Jesper Eugen-Olsen, Karina Banasik, Sisse Rye Ostrowski

**Affiliations:** 1 Department of Clinical Immunology, Copenhagen University Hospital, Rigshospitalet, Copenhagen, Denmark; 2 Department of Immunology, Aarhus University Hospital, Aarhus, Denmark; 3 Department of Immunology, Zealand University Hospital, Køge, Denmark; 4 Department of Clinical Research, Copenhagen University Hospital Amager and Hvidovre, Hvidovre, Denmark; 5 Novo Nordisk Foundation Center for Protein Research, Faculty of Health and Medical Sciences, University of Copenhagen, Copenhagen, Denmark

**Keywords:** Autoimmunity, Cytokines, Inflammation, Sleep disorders

## Abstract

Restless Legs Syndrome (RLS) is a neurological sensorimotor disorder negatively impacting sufferers’ quality of sleep and health-related quality of life. The pathophysiology of RLS is poorly understood and research focusing on the link between RLS and inflammation has been limited. Our study aimed to investigate whether chronic inflammation markers C-reactive protein (CRP) and soluble urokinase-type plasminogen activator receptor (suPAR), as well plasma levels of five different cytokine-specific autoantibodies (c-aAb), i.e. modulators of inflammation, associate with RLS in otherwise healthy individuals. CRP, suPAR and c-aAb were measured in plasma samples of participants from the Danish Blood Donor Study in 2010. Returning donors between 2015 and 2018 completed the validated Cambridge-Hopkins RLS-questionnaire for RLS assessment, resulting in datasets with RLS assessment and values for CRP (N = 3564), suPAR (N = 2546) and c-aAb (N = 1478). We performed logistic regression models using the CRP, suPAR or c-aAb as the independent variable and RLS status as the dependent variable, adjusted for appropriate covariates. Our study indicates that a high concentration of CRP is associated with RLS, while an increased probability of experiencing frequent RLS symptoms in those with an elevated plasma suPAR level appears to be mediated through lifestyle factors. We additionally report that a high titer of autoantibodies specific against the cytokine interferon-alpha was associated with RLS. Our results support the existence of links between systemic inflammation and RLS, though further RLS studies on CRP, suPAR and c-aAb in larger cohorts are warranted to confirm our findings and further reveal the hitherto underexplored links between RLS and inflammation.

## Introduction

Restless Legs Syndrome (RLS) is a neurological sensorimotor disorder with a prevalence ranging from 5 to 19% in European populations, and 5.3% in Danish blood donors^1–4^. The predominant symptom of RLS is an irresistible and persistent urge to move one's legs and either exclusively occurs or worsens during the evening and night, which in turn negatively impacts quality of sleep among sufferers^5^. RLS-associated health risk factors include obesity, smoking, high alcohol intake and low physical activity^1,4,6^. The aetiology of RLS is effectively unknown, although current accepted pathways include genetic predisposition, iron dysregulation in the central nervous system and dopaminergic dysfunction^7,8^. Systemic inflammation has also been proposed as being involved in the pathophysiology of RLS as many conditions and diseases that are highly associated with RLS also have links to inflammation^9^. Research focusing on the link between RLS and inflammation has been limited to only a few small studies, with discrepant findings. Some of these studies, for example, report an association between RLS and circulating levels of the inflammatory markers C-reactive protein (CRP)^10,11^, interleukin-6 (IL-6)^12^ and neutrophil-to-lymphocyte ratio (NLR)^13^, while others do not find associations with CRP^12,14^, IL-6^14^ and NLR^15,16^. Among RLS patients, those with increased periodic leg movements have also been found to have increased CRP levels, suggesting that severity or frequency of symptoms experienced may also associate with inflammation^17^. Serum levels of cytokines interleukin-1beta (IL-1β) and tumour-necrosis factor alpha (TNF-α) have also recently been reported as being associated with RLS. However, the study’s sample size was small (29 RLS cases and 65 controls)^12^.Therefore, further research on the link between RLS, CRP, and other inflammatory markers is warranted, especially in larger study populations. The plasma protein soluble urokinase-type plasminogen activator receptor (suPAR) is a marker for chronic inflammation (also termed low-grade inflammation) and is one such marker that has not been studied with respect to RLS. SuPAR is measurable in all individuals, and an elevated plasma suPAR level reflects immune activation and has been found to predict various health outcomes, including incident cancer, cardiovascular disease, diabetes, depression, as well as early mortality^18–20^.

Cytokines are essential signalling molecules that play significant and complex roles in both pro-inflammatory and anti-inflammatory immune responses by mediating intercellular communication between cells^21^. Importantly, cytokine-specific autoantibodies (c-aAb) are observed among both healthy and diseased individuals as naturally occurring autoantibodies capable of functional cytokine neutralization, the levels of which generally increase with higher age^22^. Their exact role, if any, is not well known, as they may both contribute to the regulation of cytokine homeostasis and play pathogenic roles in several diseases^23^.

To reveal potential links between RLS and inflammation, our exploratory study assessed whether historical measurements of inflammation-related plasma biomarkers CRP and suPAR, as well as five c-aAb, associated with RLS in blood donors from the Danish Blood Donor Study.

## Methods

### Study population

The study population consisted of participants from The Danish Blood Donor Study (DBDS), a nationwide research platform utilizing the existing infrastructure in the Danish blood banks by including blood donors when they show up to donate^24^. DBDS participants were between the ages of 18 and 67 years and were required to be generally healthy to be eligible as blood donors. Blood donors are permanently excluded from blood donation if diagnosed with chronic diseases such as diabetes, cancer, cardiovascular diseases including hypertension and statin-treated hypercholesterolemia, autoimmune diseases, hepatitis, chronic respiratory diseases, kidney diseases and metabolic diseases. Blood donors are also excluded if they are deficient in iron and if they weigh less than 50 kg. At every donation, the blood donors are also asked whether they have consulted a doctor since last donation. Upon enrolment, participants provided written informed consent, whole blood, plasma, and answered a comprehensive questionnaire, including items on height, weight and smoking status. The project was approved by the Research Ethics Committee (M-20090237) and by the Danish Data Protection Agency under the combined approval for health care research at The Capital Region of Denmark (P-2019-99), and all methods were performed in accordance with the relevant guidelines and regulations. CRP, suPAR and c-aAb were measured in plasma samples of participants from the Danish Blood Donor Study in 2010. DBDS participants between 2015 and 2018 completed the validated Cambridge-Hopkins RLS-questionnaire for RLS assessment, and therefore for this study, we identified the returning DBDS donors with both RLS data and historical measurements of either CRP, suPAR or c-aAb. This process is visualized as a timeline and flowchart in Supplementary Fig. S1.

### Chronic inflammation markers measurement

*CRP* CRP was measured in plasma samples from 18,672 DBDS participants included between March and December 2010. CRP was measured by a commercially available, high-sensitivity assay on an automated system (Ortho Vitros 5600, Ortho Clinical Diagnostics, Rochester, NY, USA) as described previously^25^. Plasma samples were stored at − 20 °C prior to analysis and thawed once prior to CRP measurement. The measuring range of the assay was 0.10–15.00 mg/L. A default value of 0.05 mg/L was assigned to samples below the lower limit of detection; no samples beyond the upper limit of detection were encountered. We used the common practice of defining chronic (i.e. low grade) inflammation as CRP levels above 3 mg/L whilst excluding participants with CRP above 10 mg/L to prevent possible cases of prevalent infection^25–27^. A total of 52,921 DBDS participants have answered the RLS questionnaire, of which 3616 of these were returning blood donors from 2010 where CRP measurements were conducted. After excluding individuals with CRP > 10 mg/L (n = 34) and missing covariate data (n = 18), the final number of participants with RLS status assessed, CRP measurements and covariate data were N = 3564. Any individuals with CRP > 10 mg/L have therefore been excluded in all analyses and therefore those classified as having “high CRP” have CRP values between 3 and 10 mg/L, compared to the other group that includes individuals with CRP less than 3 mg/L.

*SuPAR* SuPAR was measured in 14,052 DBDS participants between March and December 2010. Plasma samples were stored at − 20 °C prior to analysis and thawed once prior to suPAR measurement. Plasma suPAR levels were measured in the DBDS cohort using the CE/IVD-approved suPARnostic AUTO Flex ELISA (ViroGates A/S, Birkerød, Denmark) following the manufacturer’s instructions. The suPARnostic assay utilizes two monoclonal antibodies: a capture antibody directed towards the D_III_ subunit and a detection antibody against the D_II_ subunit. Full length suPAR (D_I_D_II_D_III_) may be cleaved into D_I_ and D_II_D_III_, and the assay captures free full length suPAR (D_I_D_II_D_III_) as well as the suPAR fragment (D_II_D_III_) but not the D_I_ fragment. The D_I_D_II_D_III_ full length suPAR molecule can bind urokinase plasminogen activator (uPA) and D_I_D_II_D_III_/uPA complexes will not be detected in the suPARnostic assay^28^. Outliers, defined as values that differed more than four times the standard deviation from the mean, were excluded (n = 45). After excluding participants with missing smoking or BMI data (n = 7), The final number of participants with suPAR levels, RLS status and covariate data available was N = 2546.

### C-aAb measurement

Five c-aAb against IL-1α (interleukin-1alpha), IL-6, IL-10 (interleukin-10), IFN-α (interferon-alpha) and GM-CSF (granulocyte macrophage colony-stimulating factor) were measured in a subset of DBDS participants, as previously described^29^, using plasma from 8919 participants collected between March and December 2010^22^. Plasma samples were stored at − 20 °C prior to analysis and thawed once prior to CRP measurement, and once more prior to c-aAb measurement. After excluding participants with missing smoking or BMI data (n < 5), 1478 participants assessed for RLS had c-aAb measurements available. High levels of c-aAb were defined as median fluorescence intensity (MFI) signals above the 99th percentile, to identify participants with c-aAb levels most likely to inhibit their target cytokines, as previously described^29^. In the RLS-c-aAb dataset, the 99th percentile cut-offs for MFI values were determined as the following: 9472 (IL-1α-aAb), 8965 (IL-6-aAb), 1096 (IL-10-aAb), 2039 (IFN-α-aAb), and 6137 (GM-CSF-aAb). The N = 1478 encompasses participants with at least one MFI value for at least one c-aAb. The number of missing MFI values observed include n = 7 (IL-1α-aAb), n < 5 (IL-6-aAb), n = 14 (IL-10-aAb), n < 5 (IFN-α-aAb), and n = 20 (GM-CSF-aAb) respectively.

### RLS assessment

RLS-status was determined using the Cambridge-Hopkins RLS-questionnaire (CH-RLSq), which is a questionnaire containing 10 items and has been validated in several population settings (diagnostic sensitivity 87.2% and specificity 94.0%)^30^. The questionnaire was translated from English to Danish using the back-translation method as previously described^4^. A total of 52,921 DBDS participants donated blood between July 2015 and May 2018 and answered the CH-RLSq for RLS assessment. The blood donor must have experienced the RLS symptoms within the past 12 months to be considered an RLS case. To be classified as an RLS case with frequent RLS symptoms (also referred to as frequent RLS cases), the blood donor must answer either “2–3 days per week”, “4–5 days per week” or “every day” to the question “In the past 12 months, how often did you experience these feelings in your legs?”. Donors with correctly completed CH-RLSq, complete covariate data and either hsCRP, suPAR, or c-aAb measurements available were used in the analysis. Supplementary Fig. S1 visualises this process as a flowchart and timeline.

### Statistical analyses

Differences between RLS cases and controls were first compared using descriptive statistics including median with interquartile ranges (IQR) for non-normally distributed quantitative variables, and count number with percentages for categorical variables. The statistical significance of these were examined using the chi-square test for categorical variables and Kruskal–Wallis rank test for continuous variables. For the c-aAb exploratory analyses, the number of RLS cases with high c-aAb was low (≤ 5) and therefore the Fisher’s exact test was used instead of the chi-square test. Logistic regression models were then used to assess the effect of the inflammation biomarkers on the probability of having RLS. Logistic regression models were then employed to assess the effect of CRP, suPAR and c-aAb on the probability of having RLS. Three models were performed: Model 0 = Crude association; Model 1 = adjusting for sex and age; Model 2 = adjustment for sex, age, smoking status and BMI. A *P*-value < 0.05 was defined as being statistically significant. All statistical analyses were performed using R (version 4.0).

## Results

### Chronic inflammation markers and RLS

For the CRP analysis, plasma CRP measurements, RLS status and covariate data were available in N = 3564 DBDS participants, of which 170 (4.8%) were characterized as RLS cases, and 17 of these reported experiencing frequent RLS symptoms (i.e. occurring 2–3 times a week or more). The proportion of females in the RLS cases group was higher than in controls (55.3% vs. 41.7%, *P* < 0.001) but there were no significant differences in age, BMI, smoking status or duration between CRP measurements and RLS CH-RLSq completion between RLS cases and controls (Table 1). For the suPAR analysis, plasma suPAR levels, RLS status and covariate data were available in N = 2546 DBDS participants, of which 126 (4.9%) were diagnosed as RLS cases, including 12 who experienced frequent RLS symptoms. As in the CRP RLS sample, the proportion of females in the RLS cases was higher than in controls (54.8% vs. 41.5%, *P* = 0.003) but there were no significant differences in age, BMI, smoking status or duration between suPAR measurements and RLS CH-RLSq completion between RLS cases and controls (Table 1).

**Table 1:** Demographic descriptive statistics of RLS cases and controls in the DBDS cohort with available CRP data (N = 3564) and the DBDS cohort with available suPAR data (N = 2546).

	DBDS cohort with CRP and RLS data (N = 3564)					DBDS cohort with suPAR and RLS data (N = 2546)				
	Controls N = 3394		RLS cases N = 170 (4.8%)		*P* value^a^	Controls N = 2420		RLS cases N = 126 (4.9%)		*P* value^a^
	N	%	N	%		N	%	N	%	
**Sex**										
Male	1978	58.3	76	44.7	** < 0.001**	1415	58.5	57	45.2	**0.003**
Female	1416	41.7	94	55.3		1005	41.5	69	54.8	
**Age**										
Years median (IQR**)**	38.9 (29.4–47.3)		40.5 (31.6–48.4)		0.245	39.1 (29.8–47.3)		40.4 (32.4–48.2)		0.312
**BMI**										
Median (IQR)	25.1 (23.0–27.7)		25.2 (22.5–28.1)		0.627	25.1 (23.1–27.7)		25.2 (22.2–28.3)		0.614
< 18.5	16	0.5	0	0	0.844	15	0.6	0	0	0.933
18.5–25	1653	48.7	82	48.2		1170	48.3	62	49.2	
25–30	1304	38.4	68	40.0		933	38.6	46	36.5	
30–35	316	9.3	14	8.2		223	9.2	13	10.3	
35–40	85	2.5	< 5	< 2.9		66	2.7	< 5	< 4	
> 40	20	0.6	< 5	< 2.9		13	0.5	< 5	< 4	
**Smoking status**										
Non-smoker	3008	88.6	149	87.6	0.470	2160	89.3	107	84.9	0.170
< 1 cigarette per day	118	3.5	< 5	< 2.9		82	3.4	< 5	< 4	
> 1 cigarette per day	268	7.9	17	10		178	7.4	15	11.9	
**Duration between donation date for hsCRP/suPAR measurement and RLS questionnaire completion**										
Years, median (IQR)	6.2 (5.6–7.0)		6.2 (5.7–7.0)		0.611	6.3 (5.7–7.0)		6.2 (5.7–7.0)		0.786

*DBDS* danish blood donor study cohort, *IQR* interquartile range.

^a^For comparison of the two groups, chi-square test was used for categorical variables and Kruskal–Wallis rank test was used for continuous variables.

Significant values are in [bold].

**Figure 1:**
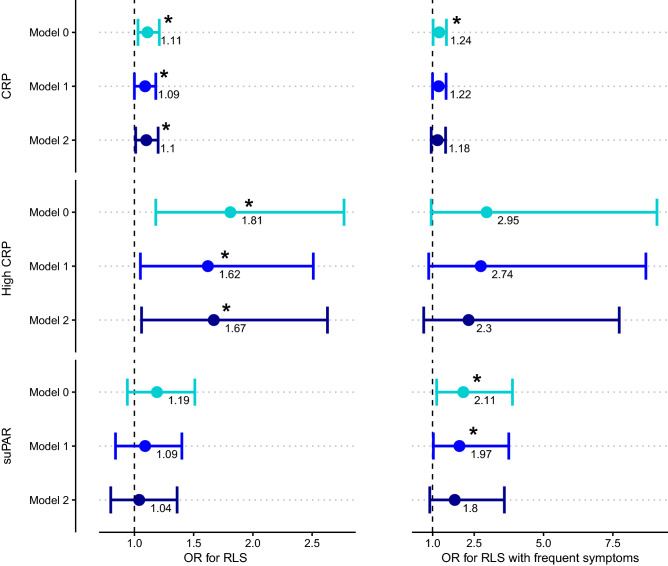
Chronic inflammation markers and association with RLS. Visualisation of the results from the logistic regression models using CRP levels (top row), CRP as a binary variable (middle row), and suPAR levels (bottom row) as the independent variable and RLS status (left column) or RLS with frequent symptoms (right column) as the dependent variable, using three different models. Model 0 = Crude association. Model 1 = adjusting for sex and age. Model 2 = adjusting for sex, age, smoking status and BMI. Data are presented as odds ratios (OR) with 95% confidence intervals, and an asterisk (*) denotes a *P* value < 0.05. OR = odds ratio. High CRP is classified as having CRP levels above 3 mg/L but below 10 mg/L.

RLS cases had higher levels of CRP than in controls (median CRP concentration [IQR (interquartile range)]: 0.74 mg/L [0.17–1.90] in RLS cases vs. 0.52 mg/L in controls [0.14–1.39]; *P* = 0.032). Similarly, we found that increased CRP levels were associated with RLS after adjusting for sex, age, smoking and BMI (OR = 1.10 [1.01–1.20] per 1 mg/L increase of CRP; *P* = 0.031) (Table 2 and Fig. 1). Increased CRP levels were also associated with RLS cases experiencing frequent symptoms in the crude logistic regression model (OR = 1.24 [1.02–1.50]; *P* = 0.033) but not after adjusting for sex, age, BMI and smoking status (OR = 1.18 [0.95–1.47]; *P* = 0.139).

**Table 2:** Chronic inflammation in RLS. Table reports the number and proportion of RLS cases (including those reporting frequent RLS symptoms) and controls with median CRP levels, high CRP levels, and the median suPAR levels in RLS cases and controls. Results from the logistic regression models are also presented using CRP and suPAR levels as the independent variable and RLS status as the dependent variable in the DBDS cohort with available CRP data (N = 3564) and suPAR data (N = 2546).

	N cases	RLS cases	Controls	Logistic regression models					
				Model 0^d^		Model 1^e^		Model 2^f^	
				OR [95% CI]	*P* value	OR [95% CI]	*P* value	OR [95% CI]	*P* value
**Median CRP [IQR] in mg/L**									
All RLS cases	170	0.74 [0.17–1.90]	0.52 [0.14–1.39]	1.11 [1.03–1.21]	**0.008**	1.09 [1.00–1.18]	**0.043**	1.10 [1.01–1.20]	**0.031**
Frequent RLS symptoms^a^	17	1.07 [0.34–2.67]	0.52 [1.14–1.39]	1.24 [1.02–1.50]	**0.033**	1.22 [1.00–1.49]	0.053	1.18 [0.95–1.47]	0.139
**High CRP**^**b**^**: N of RLS cases/controls with high CRP**^**b**^** (%)**									
All RLS cases	170	27 (15.9%)	321 (9.5%)	1.81 [1.18–2.77]	**0.007**	1.62 [1.05–2.51]	**0.030**	1.67 [1.06–2.63]	**0.026**
Frequent RLS symptoms^a^	17	1 ≤ n ≤ 5 (5.9% ≤ n ≤ 29.4%)^c^	321 (9.5%)	2.95 [0.95–9.09]	0.060	2.74 [0.86–8.69]	0.087	2.30 [0.68–7.73]	0.178
**Median suPAR [IQR] in ng/ml**									
All RLS cases	126	2.44 [2.09–2.85]	2.32 [1.97–2.83]	1.19 [0.94–1.51]	0.150	1.09 [0.84–1.40]	0.520	1.04 [0.80–1.36]	0.742
Frequent RLS symptoms^a^	12	2.72 [2.58–3.30]	2.32 [1.97–2.83]	2.11 [1.15–3.88]	**0.016**	1.97 [1.03–3.75]	**0.039**	1.80 [0.90–3.59]	0.098

*IQR* interquartile range, *OR* odds ratio, *CI* confidence interval.

^a^Cases with frequent RLS symptoms are classified as having symptoms occurring 2–3 times a week or more.

^b^High CRP is classified as having hsCRP levels above 3 mg/L but below 10 mg/L.

^c^N cases with frequent RLS symptoms and with high CRP are less than or equal to 5. Local data confidentiality protection policies prohibit the exact reporting of observations ≤ 5.

^d^Model 0 = Crude association.

^e^Model 1 = adjusting for sex and age.

^f^Model 2 = adjusting for sex, age, smoking status and BMI.

Significant values are in [bold].

We additionally created a binary CRP variable, where participants were classified as either having high CRP (CRP levels above 3 mg/L) or low CRP (below 3 mg/L). As mentioned in methods, values above 10 mg/L were excluded before the analyses to prevent possible cases of prevalent infection^25–27^. A total of 348 (9.7%) in the cohort were classified as having high CRP levels. In the RLS cases group, 15.9% had high CRP levels, compared to just 9.5% of controls (*P* = 0.006). Using a logistic regression model with RLS-status as the dependent variable, we found that donors with high CRP had higher odds for being diagnosed as an RLS case (OR = 1.81 [95%CI: 1.18–2.77]; *P* = 0.007). This association between high CRP and RLS cases remained after adjusting for sex and age (OR = 1.62 [1.05–2.51]; *P* = 0.030) and after adjusting for sex, age, BMI and smoking status (OR = 1.67 [1.06–2.63]; *P* = 0.026) (Table 2 and Fig. 1). We additionally examined the association between high CRP and frequent RLS cases. A higher proportion of frequent RLS cases were classified as having high CRP compared to controls (*P* = 0.049); however, the association was not statistically significant in the crude logistic regression model (OR = 2.95 [0.95–9.09]; *P* = 0.060) and after adjusting for sex, age, BMI and smoking status (OR = 2.30 [0.68–7.73]; *P* = 0.178) (Table 2 and Fig. 1).

Plasma suPAR levels in our samples ranged between 0.40 and 6.00 ng/ml. No statistically significant difference in plasma suPAR levels was observed between RLS cases and controls (median concentration [IQR]: 2.44 ng/ml [2.09–2.85] in RLS cases vs. 2.32 ng/ml in controls [1.97–2.83]; *P* = 0.167). Similarly, no association was found in our logistic regression models with suPAR levels as the independent variable and RLS status as the dependent variable (crude OR = 1.19 per 1 ng/ml increase of suPAR [95%CI: 0.94–1.51]; *P* = 0.150 and OR = 1.04 [95%CI: 0.80–1.36] when adjusting for sex, age, BMI and smoking status; *P* = 0.742) (Table 2 and Fig. 1). However, plasma suPAR levels were significantly higher in frequent RLS cases compared to controls (median concentration [IQR]: 2.72 ng/ml [2.58–3.30] in RLS cases vs. 2.32 ng/ml in controls [1.97–2.83]; *P* = 0.003). In the crude logistic regression model, elevated suPAR levels were associated with higher odds of being an RLS case who experiences frequent RLS symptoms (OR = 2.11 [1.15–3.88] per 1 ng/ml increase of suPAR; *P* = 0.016). This association remained statistically significant when adjusting for sex and age (OR = 1.97 [1.03–3.75]; *P* = 0.039) but not after adjusting for sex, age, smoking and BMI (OR = 1.80 [0.90–3.59]; *P* = 0.098) (Table 2 and Fig. 1).

### C-aAb and RLS

Five c-aAb were measured in a sample of N = 8919 DBDS participants in 2010, and of these, N = 1478 subsequently returned as donors between 4.6 and 8.1 years later (median 6.5 years later) and successfully completed the CH-RLS questionnaire. A total of 75 (5.1%) RLS cases were present in the dataset, of which eight (8) were frequent RLS cases. No significant differences were observed in sex, age, BMI, smoking status and duration between donation date for c-aAb measurement and RLS questionnaire completion between RLS cases and controls (Table 3). Due to the small amount of frequent RLS cases in the sample, we did not perform analyses examining c-aAb in frequent RLS cases.

**Table 3:** Demographic descriptive statistics of RLS cases and controls in the DBDS cohort with available c-aAb data and RLS data (N = 1478).

	Controls N = 1403		RLS cases N = 75 (5.1%)		*P* value^a^
	N	%	N	%	
**Sex**					
Male	785	56.0	38	50.7	0.369
Female	618	44.0	37	49.3	
**Age**					
Years, median (IQR**)**	39.4 (29.9–47.3)		40.2 (31.3–48.3)		0.532
**BMI**					
Median (IQR)	25.1 (23.0–27–8)		25.0 (22.1–27.9)		0.417
< 18.5	10	0.7	0	0.0	0.736
18.5–25	684	48.8	38	50.7	
25–30	528	37.6	30	40.0	
30–35	135	9.6	< 5	< 6.7	
35–40	38	2.7	< 5	< 6.7	
> 40	8	0.6	< 5	< 6.7	
**Smoking status**					
Non-smoker	1248	89.0	67	89.3	0.938
< 1 cigarette per day	49	3.5	< 5	< 6.7	
> 1 cigarette per day	106	7.6	5	6.7	
**Duration between donation date for C-aAb measurement and RLS questionnaire completion**					
Years, median (IQR)	6.5 (5.8–7.1)		6.5 (5.8–7.1)		0.729

*C-aAb* cytokine-specific autoantibody, *IQR* interquartile range.

^a^For comparison of the two groups, chi-square test was used for categorical variables and Kruskal–Wallis rank test was used for continuous variables.

High levels of c-aAb were defined as MFI signals above the 99th percentile. A significant difference (*P* = 0.037) was found between the proportion of individuals with high levels of IFN-α autoantibodies in RLS cases and controls (Table 4). No significant differences in the proportion of individuals classified with high c-aAb were found between RLS cases and controls for IL-1α-, IL-6-, IL-10- and GM-CSF autoantibodies.

**Table 4:** Number and proportion of RLS cases and controls with high c-aAb (IL-1α, IL-6, IL-10, IFN-α and GM-CSF -specific autoantibodies).

C-aAb	N	N cases	N controls	N cases with high c-aAb^a^	N controls with high c-aAb^a^	*P*-value^b^
IL-1α	1471	73	1398	1 ≤ n ≤ 5 (1.4% ≤ n ≤ 6.8%)^c^	13 (0.9%)	0.168
IL-6	1477	75	1402	0 (0.0%)	15 (1.1%)	1
IL-10	1464	73	1391	0 (0.0%)	14 (1.0%)	1
IFN-α	1476	75	1401	1 ≤ n ≤ 5 (1.3% ≤ n ≤ 6.7%)^c^	12 (0.9%)	**0.037**
GM-CSF	1458	73	1385	0 (0.0%)	15 (1.1%)	1

^a^High c-aAb is classified as having a c-aAb level above the 99th percentile of the given c-aAb dataset.

^b^Statistical significant difference tested through Fisher’s exact test.

^c^The number of RLS cases with high c-aAb (either for IL-1α or IFN-α) is between 1 and 5. Local data confidentiality protection policies prohibit the exact reporting of observations ≤ 5.

Significant values are in [bold].

As there was a significant difference in IFN-α autoantibodies between RLS cases and controls, we performed logistic regression models using high IFN-α-autoantibodies as the independent variable and RLS status as the dependent variable. Individuals with high IFN-α-autoantibody levels (above the 99th percentile) were over four times as likely to have RLS when compared to those with low IFN-α-autoantibody levels in the unadjusted regression model (OR = 4.82 [95%CI: 1.33–17.47]; *P* = 0.017). The association between high IFN-α-autoantibody levels and RLS status remained after adjusting for sex and age (OR = 4.70 [1.29–17.08]; *P* = 0.019), and in model 2 which adjusted for sex, age, BMI and smoking status (OR = 4.78 [1.28–17.80]; *P* = 0.020).

## Discussion

We examined whether historical plasma measurements of the chronic inflammation markers hsCRP and suPAR, as well as five c-aAb, were associated with RLS in otherwise healthy Danish blood donors. We report associations between elevated levels of CRP and RLS, and between suPAR and RLS with frequent symptoms (though dependent on smoking and BMI), and high IFN-α autoantibody levels in individuals with RLS. These intriguing findings support the notion that links between RLS and systemic inflammation exist.

Increased CRP levels in plasma were associated with RLS even after adjusting for sex, age, BMI and smoking status. This supports findings from a study in which RLS associated with higher serum CRP levels (Olgun Yazar et al.^10^: 197 RLS cases, 193 controls), and a study of haemodialysis patients in which RLS cases also had significantly higher serum CRP levels (Higuchi et al.^11^: 33 RLS cases, 124 controls). However, other studies have reported no association between RLS and CRP, including a similarly sized study as ours (Benediktsdottir et al.^14^: 205 RLS cases, 1,139 controls vs. DBDS: 170 RLS cases, 3,394 controls) and a small study (Uslu et al.^12^: 29 RLS cases, 65 controls). There are several differences between our study and the study by Benediktsdottir et al. (2010) that may explain the discrepant finding. Benediktsdottir et al. (2010) pooled two separate population cohorts from Iceland and Sweden (we used a uniform population of healthy blood donors) and they also excluded adults under age 40 (DBDS median age in CRP sample: 39.0 years; range: 18–61 years). Additionally, Benediktsdottir et al. (2010) measured CRP in serum and not plasma. To our knowledge, our study is the first to investigate RLS in relation to plasma CRP as a binary variable using this cut-off, and also the first in inflammation-focused studies to identify RLS cases by using the validated CH-RLS questionnaire in contrast to the International Restless Legs Syndrome Study Group (IRLSSG) questionnaire which was used in the other studies. It is likely, though unknown, whether these listed differences may explain our discrepant results. Nevertheless, our results indicate that an association exists between RLS and high CRP levels in blood donors.

We also investigated the chronic inflammation marker suPAR and RLS. No significant difference in plasma suPAR levels was observed between RLS cases and controls in our sample; however, we report an association between suPAR levels and RLS cases experiencing frequent RLS symptoms. The association remained after adjusting for sex and age, but not when additionally adjusting for smoking status and BMI. Smoking is strongly associated with elevated suPAR levels, and suPAR can be lowered by smoking cessation^31^. Furthermore, an unhealthy diet, inactive lifestyle and obesity have substantial impacts on suPAR levels in the general population^20,32,33^. Our results therefore indicate that suPAR may associate with severe cases of RLS, although the association seems to be driven by smoking and high BMI, which also induces high suPAR levels. It must be noted that the association relies on a small sample of RLS cases that report experiencing frequent RLS symptoms. Likely due to the healthiness of the blood donors, only 12 Danish blood donors in our suPAR-RLS dataset reported having frequent RLS symptoms, which corresponds to only 9.5% of RLS cases, and 0.5% of the total dataset. With only 12 frequent RLS cases in the suPAR sample, the model adjusting for sex, age, smoking and BMI may be overfitted. However, despite the limited number of severe RLS cases in our cohort, to our knowledge this is the first study to investigate suPAR and RLS and therefore these results may inspire further studies examining the relationship between RLS and suPAR in larger cohorts. It is noteworthy that high CRP associated with RLS cases but not in those with frequent RLS symptoms, whereas the reverse was true for suPAR. This may simply be due to the limited number of donors with frequent RLS symptoms, thereby restricting the statistical power, however it is also possible that this is due to suPAR and CRP reflecting different aspects of chronic inflammation. It has been suggested that CRP is associated with anthropometric measures of inflammation, while suPAR is linked to cellular and vascular inflammatory processes, such as endothelial dysfunction and atherosclerosis^34^. In contrast to CRP, suPAR also differs in that it is more stable, with minimal circadian changes in plasma suPAR^35–37^. Despite the differences between CRP and suPAR, our results generally suggest that chronic inflammation is associated with RLS, with plasma CRP levels above 3 mg/L being associated with higher odds for RLS in healthy blood donors, whereas an elevated plasma suPAR level increases the probability of experiencing frequent RLS symptoms, linked to lifestyle factors.

Of the five c-aAb measured in the DBDS, we report an association between RLS and IFN-α autoantibodies. Individuals with high IFN-α-autoantibody levels (above the 99th percentile) were over four times as likely to have RLS when compared to those with low IFN-α-autoantibody levels. IFN-α plays a significant role in the pathophysiology of systemic lupus erythematosus (SLE), a classic chronic inflammatory autoimmune disease which dysregulates the innate and adaptive immune system^23^. Elevated levels of IFN-α autoantibodies have been shown to have a protective effect in SLE, thereby capable of lowering disease severity^23,38,39^. Several smaller studies have found that the prevalence of RLS is higher in SLE patients than in controls^40–42^, although whether they are caused by a common pathophysiological pathway involving IFN-α is unknown. A case report by LaRochelle and Karp^43^ documented a patient that developed RLS after IFN-α therapy for chronic hepatitis C. Between 50 and 75% of patients treated with recombinant IFN-α develop anti-interferon antibodies^44^. There is a possibility that both therapy-induced and natural anti-interferon antibodies cause a disturbance in the interferon system, which can lead to pathological changes affecting the CNS. In addition to RLS, several other case reports have found adverse side effects on the nervous system after IFN-α therapy^45^, including involuntary facial movements and weakness^46,47^ and sensorimotor polyneuropathy^48^, further supporting either IFN-α or anti-IFN antibodies’ potential of affecting the CNS and specifically causing sensorimotor neurological disorders. To our knowledge, no study to date has examined the effect of IFN-α and IFN-α autoantibodies in relation to RLS. Further RLS studies on IFN-α and IFN-α autoantibodies in larger cohorts are needed to confirm and extend our findings.

To our knowledge, our study is the first to investigate suPAR levels and c-aAb in individuals with RLS. A clear strength of our study is our uniformly healthy study population, as blood donors are thoroughly screened at every visit to the blood bank and are required to be healthy to be eligible as blood donors, which in turn reduces the presence of comorbidities that may confound true suPAR- and c-aAb-RLS associations. Danish blood donors are also excluded if they are deficient in iron and we have previously shown that RLS is not associated with a reduced ferritin level in our DBDS population^1^. Additionally, although inflammation is known to induce the synthesis of the iron-regulatory protein hepcidin^49^, we have previously shown that in our DBDS population, there is no association between increased hepcidin levels and RLS^50^. However, we acknowledge that a notable limitation of the study is the duration between the donation date of biomarker measurement, and the date of RLS status assessment (via the completion of the CH-RLS questionnaire). A cross-sectional study with RLS status and biomarker measurement taken simultaneously is preferable however not currently possible in our cohort. It is not known whether the delayed determination of RLS status may have impacted the results, however no significant differences were observed between the duration of donation and RLS questionnaire completion between cases and controls in the three samples. To ensure our analyses were less sensitive to CRP variability, we converted CRP measurements into a binary variable that represents chronic inflammation rather than an acute inflammatory state. Furthermore, as mentioned previously, suPAR is a stable protein with minimal variations and measurements have been shown to be correlated in individuals across 5 and 7 years^33,51^. C-aAb are also stable over time, especially for individuals with high-titer levels^52^. However, we also acknowledge the stringent 99th percentile cut-off in the c-aAb analyses as being a limitation as it restricted the number of RLS cases available for the analyses. As a result, the adjusted models examining IFN-α-aAb with respect to RLS may have been overfitted. Larger, cross-sectional studies or studies with known baseline RLS status and follow-up data are therefore required to validate the associations found in our study between RLS and CRP, suPAR and IFN-α autoantibodies.

Our study focused on investigating links between several inflammation-related markers and RLS. In addition to inflammation and the three widely-accepted pathways (Iron dysregulation, CNS dopaminergic dysfunction and genetic predisposition), hypoxia has also been suggested as possibly being involved in RLS pathophysiology^53,54^. Several small studies have shown that hypoxic pathways may be involved in RLS pathophysiology^55–57^ and it may also be possible that there is a connection between inflammation and hypoxia in respect to the development of RLS. Hypoxic-inducible factors are essential regulators of inflammation^58^ and have previously been associated with RLS^55^. A small proteomic analysis study also reported that eight proteins out of 492 tested were differentially expressed in RLS patients, of which all eight proteins were related to inflammation^59^. Their enrichment pathway and network analysis revealed indirect connections to proteins related to hypoxic pathways. Future studies may therefore investigate this potential connection between hypoxia and inflammation in RLS further. In conclusion, using Danish blood donors, our study indicates that a high concentration of CRP is associated with RLS, while an elevated plasma suPAR level, linked to lifestyle factors, increases the probability of experiencing frequent RLS symptoms. We additionally report that a high titer of IFN-α autoantibodies was associated with RLS. Our results support the existence of links between systemic inflammation and RLS, though further RLS studies on CRP, suPAR and c-aAb in larger cohorts and in RLS patients with higher frequency and severity are warranted to confirm our findings and further reveal the hitherto underexplored links between RLS and inflammation.

## Supplementary Information

Supplementary Information.

## Data Availability

For information on further access to data included in the study, please contact Sisse Rye Ostrowski from the Danish Blood Donor Study (Sisse.Rye.Ostrowski@regionh.dk).

## Code availability

We used the publicly available software R (version 4.0) to analyze data and create plots.
