# Supplementary material for: Chronic inflammation markers and cytokine-specific autoantibodies in Danish blood donors with restless legs syndrome
Source: Sci Rep. 2022 Jan 31;12:1672. doi: 10.1038/s41598-022-05658-1 (PMC8803845; doi:10.1038/s41598-022-05658-1)
Supplement: Supplementary file 1 — Supplementary Information. [file 41598_2022_5658_MOESM1_ESM.pdf]

SUPPLEMENTARY INFORMATION

Chronic Inflammation Markers and Cytokine-specific Autoantibodies in Danish Blood Donors with Restless Legs Syndrome

Joseph Dowsett, Maria Didriksen, Jakob Hjorth von Stemann, Margit Hørup Larsen, Lise Wegner Thørner, Erik Sørensen, Christian Erikstrup, Ole Birger Pedersen, Morten Bagge Hansen, Jesper Eugen-Olsen, Karina Banasik, Sisse Rye Ostrowski

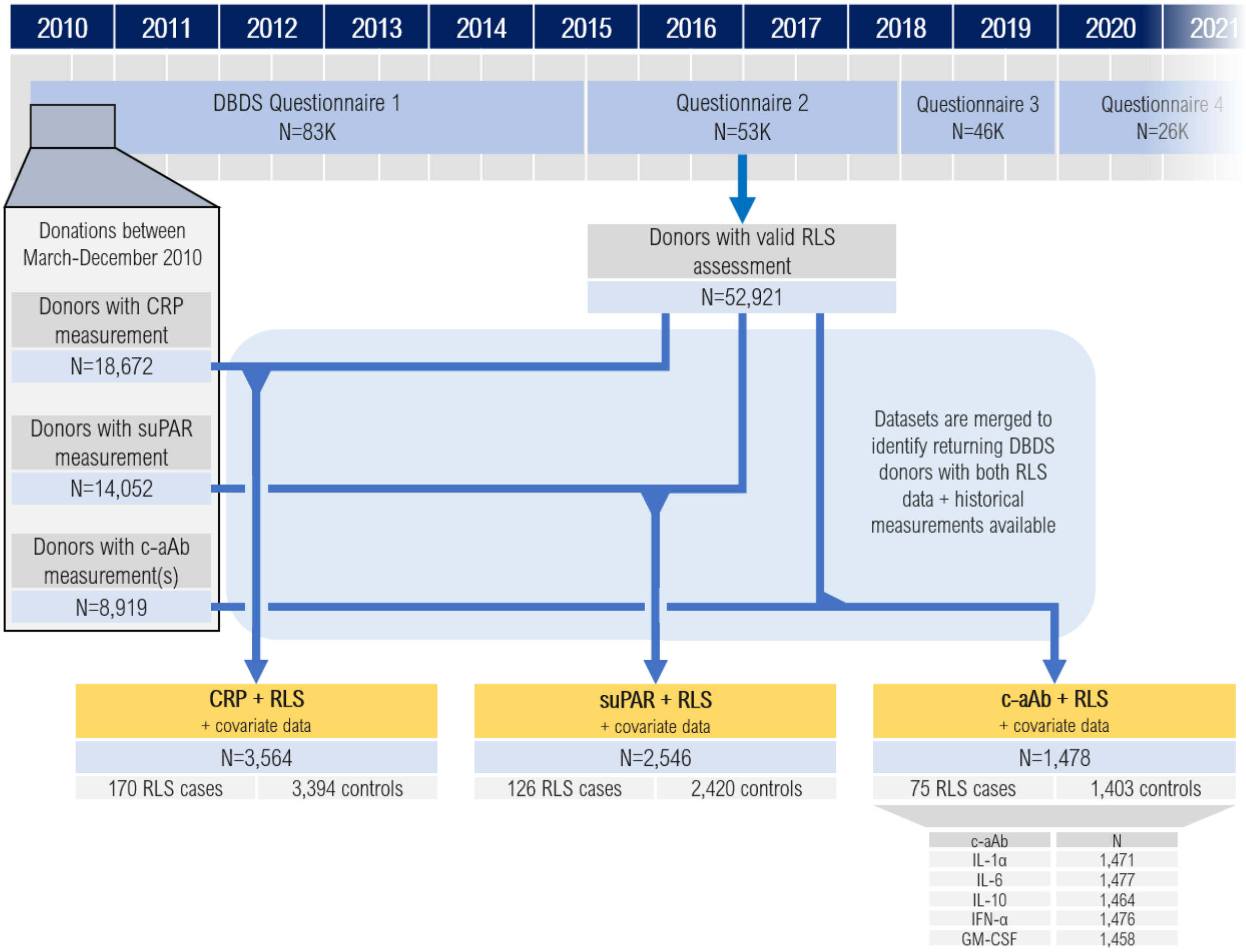

Supplementary Fig. S1: Timeline and flowchart visualising how the final three datasets were determined. The yellow boxes signify the final datasets. After the successful merging of datasets, the number of participants with CRP measured and RLS assessment was 3,564 donors. For suPAR, returning donors who had previously had suPAR measured in 2010 and had RLS assessed was N=2,546. For cytokine-specific autoantibodies (c-aAb), the number of donors with c-aAb measurements and RLS assessment was N=1,478.
